# Transrectal Ultrasound Guided Prostatic Biopsy and its Complications: A Descriptive Cross-sectional Study

**DOI:** 10.31729/jnma.4820

**Published:** 2020-01-31

**Authors:** Robin Joshi

**Affiliations:** 1 Department of Urology, Kathmandu Medical College and Teaching Hospital, Sinamangal, Kathmandu, Nepal

**Keywords:** *biopsy*, *prostate-specific antigen*, *magnetic resonance imaging*

## Abstract

**Introduction:**

Transrectal ultrasound of prostate provides better visual for biopsy. Transrectal ultrasound guided prostate biopsy is usually performed in men with an abnormal digital rectal examination, and elevated prostate specific antigen (>4ng/ml) or prostate specific antigen velocity (rate of prostate specific antigen change) i.e., >0.4-0.75ng/mI/year. The aim of the study is to find out the complications of transrectal ultrasound guided prostatic biopsies.

**Methods:**

This descriptive cross-sectional study was done among 50 patients who transrectal ultrasound guided prostatic biopsies in a tertiary care hospital, from July 2017 to July 2019 after receiving ethical approval from the Institutional Review Committee of Kathmandu Medical College and teaching hospital. Convenient sampling was done. All patients were informed about the potential benefits and risks of the transrectal ultrasound guided prostate biopsy and patients signed an informed written consent form. Statistical analysis was done by using Statistical Package for Social Sciences version 16.

**Results:**

Mean prostate specific antigen was 34.571 and mean weight of prostate was 44.6gm. Moderate to severe pain was experienced by 15 (30%), 2 (4%) had hematuria with fever accounting for 3 (6%) patients. All were managed conservatively with no mortality related to the procedure and complication. Three patients was positive for malignancy on re-biopsy.

**Conclusions:**

Transrectal ultrasound guided biopsy of prostate is a pioneer experience in Nepal. It has proved to be an useful tool of diagnosis of suspected carcinoma of Prostate. Use of neurovascular block may reduce the pain during the procedure.

## INTRODUCTION

Prostate cancer is the second leading cause of male cancer death in Europe and North America. Opportunistic or population screening programme using the prostate specific antigen (PSA) test have been introduced to detect localized cancer stages that may progress to advanced disease.

Consequently, many new biopsy protocols have been proposed with respect to their main features ‘number of biopsy cores’ and ‘biopsy. These days 10-12 core biopsy is advised^1,2^. Prostate imaging-reporting and data System scoring on Magnetic Resonance Imaging (MRI) may point out the presence of carcinoma. Prostate imaging-reporting and data system scoring on MRI may point out the presence of carcinoma.Targeted biopsy with cognitive magnetic resonance imaging and suspicious hypoechoic lesions on transrectal ultrasound coupled with standard systematic prostate biopsy has shown to increase detection rate of prostate cancer.^1,10^

The aim of the study is to find out the results of Transrectal Ultrasound (TRUS) guided prostatic biopsies.

## METHODS

This descriptive cross-sectional study was done among 50 patients who transrectal ultrasound guided prostatic biopsies in a tertiary care hospital, from July 2017 to July 2019 after receiving ethical approval from the Institutional Review Committee of Kathmandu Medical College and teaching hospital (KMCTH). Convenient sampling was done. All patients were informed about the potential benefits and risks of the percutaneous transrectal ultrasound and patients signed an informed written consent form. Statistical analysis was done by using Statistical Package for Social Sciences version 16.

Convenient sampling was done and the sample size was calculated using the formula,

$$\begin{array}{ll} \text{n} & \text{= }{\text{Z}}^{\text{2}}\times \text{(p}\times \text{q)}/{\text{e}}^{2}\\ & \text{= 1}{\text{.96}}^{\text{2}}\times \text{0.5}\times \text{(1}-\text{0.5)}/\text{0}{\text{.14}}^{\text{2}}\\ & \text{= }49 \end{array}$$

where,

n = required sample size
p = prevalence of study, 50%
q = 1-p
e = margin of error, 14%
Z = 1.96 at 95 % CI

The calculated minimum sample size was 49, but the total sample taken was 50.

The Examinations were carried out with Ultrasonic Transducer using the transrectal dual-plane transducer with a mean frequency of 7.5MHz. Per rectal Examination and biopsies were performed in the B- Mode. The biopsy was performed using an automated biopsy gun using 18 - gauge needle and cutting length of 22mm. Patient was placed in Sims position and perianal region was disinfested with povidine iodine solution. Anal pack with Povidine soaked 2% lignocaine gel was kept for 5 minutes.

Opiod analgesics (Tramadol-50mg) with antiemetic Ondansetron (4mg) was given Intra venously. One gram Ceftriaxone was given as prophylactic antibiotics. Core biopsy, 6-12 was performed, from right upper lateral, right upper medial, left upper medial, left upper lateral, right middle lateral, right middle medial, left middle medial, left middle lateral, right lower lateral. Right lower medial, left lower medial, left lower lateral. Targeted biopsies are also centered on prostate in MRI suspicous area. The specimen were sent in 10% formalin solution. Patient was kept under strict observation for 2 hours and was discharged with oral medication and to follow up if any complication.

## RESULTS

Thirty five (70%) of the patients experienced mild tolerable pain with15 (30%) patients had moderate to severe pain (Table 1) . Only 1(2%) of the patient have had rectal bleeding for more than 2 days and 2(4%) patients had hematuria for more than 1 day. Three (6%) of the patients had fever of more than 38.5 degree centigrade and was admitted for observation. Septicemia or urosepsis was not seen in any of the patients. Other complication like hematospermia, prostatitis and epidydimitis were not seen. Ten (20%) patients experienced acute retention of urine and were managed conservatively. A total of 6(12%) patients had complications requiring admission for observation.

**Table 1:** Complications of transrectal ultrasound guided prostatic biopsies.

Complications	n (%)
Mild tolerable pain	35 (70%)
Moderate to severe pain	15 (30%)
Rectal bleeding	1 (2%)
Hematuria	2 (4%)
Fever	3 (6%)
Acute retention of urine	10 (20%)
Prostatitis	-
Epidydimitis	-

Forty (80%) cases was found to have positive for malignancy. Seven (14%) had normal or prostatitis on HPE. Three (6%) patients underwent rebiopsy for persistently raised PSA and abnormal digital rectal examination (DRE). All were positive for malignancy on rebiopsy.

Fifty patients with mean age of 67.84 and the age range 52-87 years were studied. All patients underwent TRUS guided prostate biopsy for the suspicion of carcinoma prostate based on abnormal DRE and /or raised PSA with the support of MRI of prostate. MRI prostate was done by the affording patients only. The mean PSA was 39.571 with average mean weight of 44.6gms.

## DISCUSSION

Since the mid-1980s, TRUS biopsy has been used to diagnose prostate cancer .TRUS guided biopsy was started at KMCTH from 2014. The advent of prostatic biopsy via a transrectal approach using ultrasiund served as a welcomed replacement for the previous blind approach, which was challenging for the practitioner to get the proper core from the suspected area in prostate and painful for the patient.^1,8^ Current guidelines recommend prostatic biopsy for all patients with elevated serum prostate-specific antigen (PSA) or abnormal prostate morphology on digital rectal exam. Standard practice for transrectal ultrasound (TRUS)-guided biopsy involves the utilization of 1012 core needle 18 gauge biopsies; however, there is not an established ideal number of biopsies.^1–3^ Extended(saturaion) 22 core biopsies have been found to improve the concordance of Gleason scores between prostatic biopsies and prostatectomy.^3,8^ New advances in technology, including contrast-enhanced transrectal ultrasound (CE-TRUS) and real-time sonoelastography(RTE), have shown promise, allowing for targeted biopsies and improving detection.^4^

MRI-TRUS fusion techniques include (a) in-bore MR- guided biopsy (b) cognitive registration, and (c) software registration-based fusion. In-bore MR-guided biopsy uses pre-biopsy MRI images to identify lesions of interest. Cognitive registration entails the usage of a pre-biopsy MRI to create a map of the prostate. At the time of biopsy, a typical 2D TRUS is obtained and prebiopsy MRI helps in creating a tentative image for the biopsy and targeted biopsy on the suspicious lesions seen on MRI. A live guidance of the biopsy, known as “tracking”, is thus created.^5^ The MRI-TRUS fusion technique is the newest of common techniques to detect prostate cancer (PCa). It holds value for patients that have newly detected elevated levels of prostate- specific antigen (PSA). MRI can be used now to assess risk, increase accuracy in evaluation of tumor margins, and also guide the biopsy portion of PCadetection. It has shown to reduce the number of biopsies done on patients that have low-grade PCa, reducing the overall diagnoses of low-grade prostate cancers, and increasing the detection of the intermediate and highrisk subgroups of patients compared to traditional modalities.^5,6^ Also been found to be more accurate in biopsy targeting.^7,8^

The targeted MRI/TRUS fusion-guided biopsy is an accurate method of diagnosis of prostate cancer, especially in patient with previous negative TRUS guided prostate biopsy and suspicious prostate cancer.^9^Targeted biopsy with magnetic resonance imaging and hypoechoic lesions on transrectal ultrasound has found to increase prostate cancer detection rate. The detection ability for prostate cancer was significantly better for systematic prostate biopsy than for targeted TRUS prostate biopsy orcognitive magnetic resonance imaging-targeted biopsy (MRI-TBx) alone.^10^

TRUS guided biopsy of prostate was started in 2014 at KMCTH .We have included 50 cases with high diagnostic value and less morbidity from the period of July 2017 to July 2019. Large number of cases are required to assess the morbidity and outcome of the procedure. Prebiopsy cognitive MRI with TRUS guided biopsy were useful tools in detecting suspicious lesions and systematic TRUS biopsy with targeted biopsy on suspicious lesions yielded more positive results.

In our study fifty adult patients with the mean PSA of 39.571 (range 2.75-448.99), 40 (80%) cases was found to have positive for malignancy and 6 % on re-biopsy .Majority of the patients did not experienced severe complication. About 12 % had complications who needed admission for observation and were successfully managed conservatively. Use of injector for neurovascular bundle (NVB) block will further decrease or nullify pain. Few patients needed IV sedation to decrease the pain. Rectal bleeding and hematuria was managed conservatively. None of the patients had septicemia except for fever which was managed conservatively.

## CONCLUSIONS

The relative complication rates were very low and there was no readmission for infection or sepicemia. Neurovascular block may reduce the pain during the procedure. TRUS guided biopsy of the prostate may be considered a standard method for the diagnosis of prostatic cancer.

## Conflict of Interest

**None.**
